# Maladjustment to Academic Life and Employment Anxiety in University Students with Irritable Bowel Syndrome

**DOI:** 10.1371/journal.pone.0129345

**Published:** 2015-06-17

**Authors:** Jun Tayama, Naoki Nakaya, Toyohiro Hamaguchi, Tatsuo Saigo, Atsushi Takeoka, Toshimasa Sone, Shin Fukudo, Susumu Shirabe

**Affiliations:** 1 Graduate School of Education, Nagasaki University, Nagasaki, Japan; 2 Center for Health and Community Medicine, Nagasaki University, Nagasaki, Japan; 3 Tohoku Medical Megabank Organization, Tohoku University, Sendai, Japan; 4 Department of Occupational Therapy, School of Health and Social Services, Saitama Prefectural University, Saitama, Japan; 5 Unit of Preventive Medicine, Graduate School of Biomedical Sciences, Nagasaki University, Nagasaki, Japan; 6 Department of Rehabilitation, Faculty of Health Science, Tohoku Fukushi University, Sendai, Japan; 7 Department of Behavioral Medicine, Tohoku University Graduate School of Medicine, Sendai, Japan; National Center of Neurology and Psychiatry, JAPAN

## Abstract

The present study tested our hypothesis that university students with irritable bowel syndrome (IBS) may experience less satisfactory academic lives than those of students without IBS. We also verified the hypothesis that university students with IBS might have higher employment anxiety than students without IBS might. We conducted a cross-sectional study of 1,686 university students. Presence or absence of IBS was assessed via the Rome III Questionnaire. Two original items were used to evaluate academic life. The prevalence rates of IBS with diarrhea, IBS with constipation, mixed IBS, and unsubtyped IBS in the study population were 5%, 2%, 10%, and 3%, respectively. Regarding academic life, the proportions of participants who experienced maladjustment and employment anxiety were 29% and 50%, respectively. After adjusting for age, sex, and faculty, the odds ratios for maladjustment and employment anxiety were significantly higher in students who screened positively, relative to those who screened negatively, for IBS (OR, 1.62; 95% CI, 1.24–2.21; OR, 2.16; 95% CI, 1.68–2.81, respectively). In conclusion, maladjustment and anxiety over future employment were higher in university students with IBS relative to those without.

## Introduction

Irritable bowel syndrome (IBS) is a functional gastrointestinal disorder that is not associated with major organic disease [1, 2]. The prevalence of IBS is approximately 5–11% in developed countries [3]. The characteristic pathophysiological features of IBS are dysmotility of the lower gastrointestinal tract [4], visceral hypersensitivity [5], and psychological abnormalities [6]. These symptoms exert a significant impact on various aspects of life in individuals with IBS [7–10]. Regarding economic issues, the health care costs incurred by IBS patients are high relative to those incurred by individuals without the condition [7, 9]. In epidemiological studies, quality of life (QOL) in adult patients with IBS has been shown to be low [7–10]. In a cross-sectional study of 257 adult patients with IBS, health-related quality of life was shown to be lower than that observed in healthy individuals [7].

Adolescence is a period in which onset of IBS is common [11–14]. Until the early teenage years, the prevalence of IBS is relatively low but increases rapidly from the late teens onward [11]. In an epidemiological survey of 10,000 Japanese individuals aged 20 years or over, it was shown that the prevalence of IBS was highest in participants in their 20s (14% for men and 22% for women); morbidity was reduced in participants aged 30 years or over [14]. In a cross-sectional study of 1,086 university students in Japan, using the ROME-II modular questionnaire [15], the prevalence of IBS was 19% overall, or 17% for men and 20% for women [13]. Similarly, in another cross-sectional study of 557 university students in Japan, the prevalence, according to IBS screening using the ROME-III questionnaire, was high at 26% overall [12]. These findings suggest that the prevalence of IBS in university students is high and comparable to that observed in Japanese individuals in their 20s [11–14]. These findings suggest that the prevalence of IBS is very high in young people.

In employees with IBS, various issues occur with respect to the execution of business activities. In some previous studies, IBS symptoms were shown to cause poor performance at work [16, 17, 18]. This evidence suggests that adult workers with IBS experience performance-related difficulties at work. This performance degradation may also occur in university students with IBS. In a cross-sectional study involving 597 medical students, researchers showed that living in a dormitory while at school and emotional stress were associated with increased IBS prevalence in university students [19]. This evidence suggests that academic life may be unsatisfactory, and employment anxiety regarding health concerns is higher in students with IBS than in those without the condition. However, the issues surrounding maladjustment to academic life and employment anxiety in university students with IBS are unclear. By examining the association between the symptoms of IBS and academic life, the support needs of students with IBS and policies suitable for addressing these issues may become apparent.

In this study, we tested the following hypotheses in university students.

1. The academic lives of university students with IBS will be less satisfactory than those of students without IBS.
2. Employment anxiety will be higher in students with IBS relative to students without IBS.

If the results reveal that academic life is unsatisfactory, and employment anxiety regarding health concerns is higher in students with IBS than that observed in those without the condition, our data would suggest a need for psychological intervention as a method of meaningful assistance for students with IBS.

## Materials and Methods

### Participants

**Fig 1:**
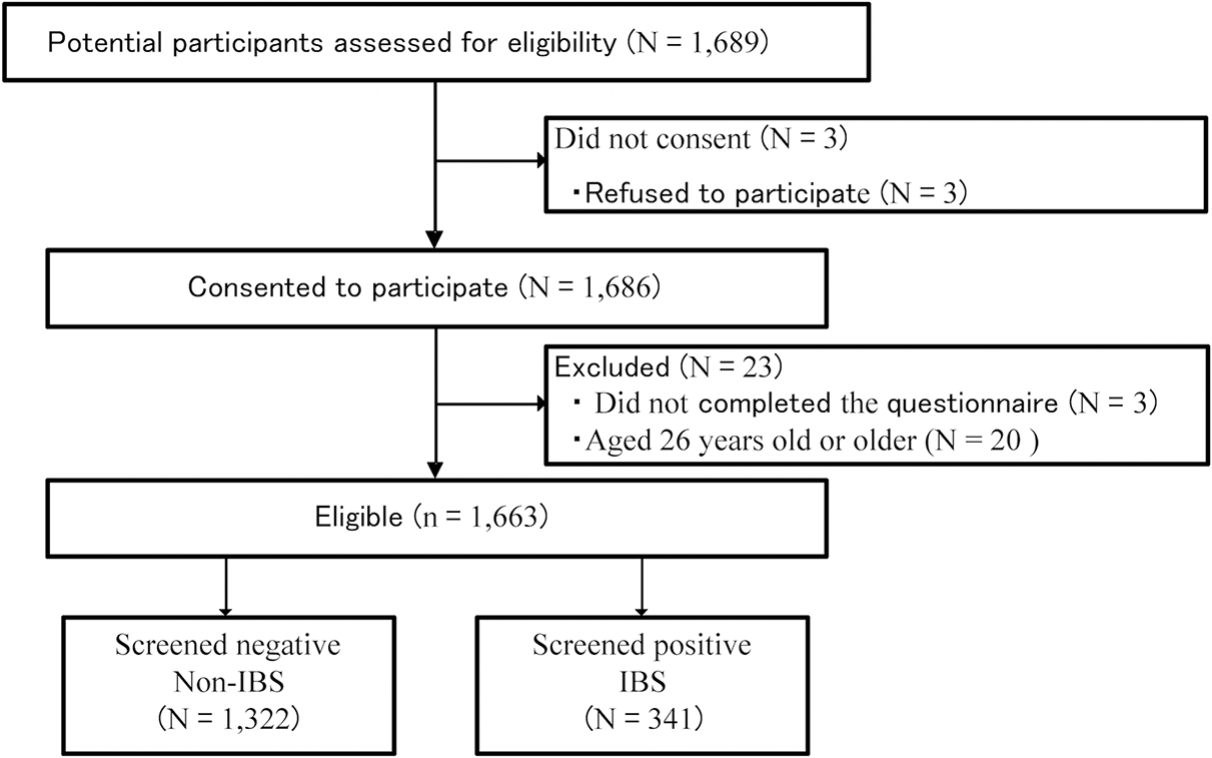
Study flow. We conducted a cross-sectional study of 1,686 university students. Statistical analysis was performed using the data collected from 1,663 participants. Of these 1,663 participants, 341 (21%) were diagnosed with IBS.

The study was conducted between May and December 2013. A total of 1,689 potential participants were assessed for eligibility (Fig 1). Three students refused to provide consent for participation in the study or use of their data for research. We therefore conducted a cross-sectional study of 1,686 university students. Three students did not respond to the questionnaire. In accordance with a previous study [20], we defined the participants in this study as young adults aged 18–25 years. Therefore, data collected from 20 students aged 26 years or older were excluded. Data collected from 1,663 participants were included in the statistical analysis. Of these 1,663 participants, 341 (21%) were diagnosed with IBS based on responses to the IBS screening questionnaire described below.

### Measurements

#### Rome III diagnostic questionnaire

Rome III criteria are widely used in the diagnosis of IBS [21, 22]. These criteria include recurrent abdominal pain or discomfort (on at least 3 days per month in the previous 3 months) associated with 2 or more of the following: 1) improved by defecation, 2) onset associated with a change in stool frequency, and 3) onset associated with a change in form (appearance) of stool. Use of the Rome III criteria can reduce the number of heterogeneous IBS patients diagnosed, without performing unnecessary examinations [21, 23]. Therefore, because the Japanese Society of Gastroenterology strongly recommends the use of the Rome III criteria [24], they were used in the questionnaire to classify participants as either IBS-positive or IBS-negative.

#### Academic life

Two original items formed an academic life questionnaire, which was used to evaluate academic life and included the following questions: 1) Maladjustment: “Can you focus all of your energy toward your academic life?” 2) Employment anxiety: “Are you concerned about whether you will maintain good psychosomatic health when you are employed in the future?” We defined employment anxiety as anxiety over health concerns in future employment. Each question required a response of either yes or no. This questionnaire was only available in Japanese, and its validity and reliability were evaluated. Maladjustment score was significantly correlated with grade point average in these participants (ρ = 0.18, p < 0.01). Test-retest reliability estimates showed that, two weeks after testing, the interclass coefficient was 0.76.

### Procedure

The questionnaire survey was presented in a classroom during an academic session. We explained the procedures and general purpose of the research and provided both written and verbal explanations of the intended use of the data. In addition, we explained that individuals who did not consent to study participation would be placed at no disadvantage whatsoever. We only analyzed data obtained from students who had agreed to participate.

### Data analysis

Means (Ms) and standard deviations (SDs) were calculated for all data. Participants were divided into 2 groups according to IBS status (IBS+ or IBS-). Multivariate logistic regression was conducted, with (good or bad) academic life used as the dependent variable. Both crude and adjusted odds ratios (ORs) and 95% confidence intervals (CIs) were calculated. There were 6 faculty categories (education, economics, medicine, dentistry, pharmacy, engineering, environmental science, and fisheries), for which we formulated 6 representative binary variables (coded 1 = yes and 2 = no); these were included in the regression analysis. The group that responded with positive evaluations in questions regarding academic life served as the reference group. In this study, the sample size was calculated according to the results of a previous study [25]. The proportions of participants with anxiety were 75% and 60% in the IBS and non-IBS groups, respectively, and the ratio of IBS to non-IBS participants was 1:8. Setting a β level of 0.20 and an α level of 0.05 in the two-tailed test, we calculated a minimum sample size of 789. As the study included 1,686 participants, the sample was sufficiently large. The significance threshold was 0.05. JMP ver. 10.0 (SAS Institute Inc.) was used for all statistical analyses.

### Ethics

The study protocol was approved by the Ethics Committee of Nagasaki University (no.12053008). All participants provided their written and verbal informed consent to participate in this study.

## Results

Participants’ demographic data and reference values for healthy individuals, which were established in a previous study, are shown in Table 1 [12, 25, 26]. The prevalence rates of IBS with diarrhea (D-IBS), IBS with constipation (C-IBS), mixed IBS (M-IBS), and unsubtyped IBS (U-IBS) in the study population were 5% (95% CI, 4–6), 2% (95% CI, 2–3), 10% (95% CI, 9–12), and 3% (95% CI, 2–4), respectively. Regarding academic life, the proportions of students who experienced maladjustment and employment anxiety were 29% and 50%, respectively.

**Table 1:** Demographic Data and Reference Values.

Variables	All participants	Reference values	
	(N = 1,663)		
	(95% CI)	(general population (95% CI))	
Sex (male (%))	61 (59–63)	59 (54–64)	Saigo, T. et al.^[^ ^21^ ^]^
Age	19 ± 1 (19–19)	-	-
IBS^+^ (%)	21 (19–23)	26 (22–30)	Tayama, J. et al.^[^ ^12^ ^]^
D-IBS (%)	5 (4–6)	4 (-)	Kubo, M. et al.^[^ ^22^ ^]^
C-IBS (%)	2 (2–3)	3 (-)	Kubo, M. et al.^[^ ^22^ ^]^
M-IBS (%)	10 (9–12)	3 (-)	Kubo, M. et al.^[^ ^22^ ^]^
U-IBS (%)	3 (2–4)	4 (-)	Kubo, M. et al.^[^ ^22^ ^]^
Faculty (%)			
Education	14 (13–16)	-	-
Economics	23 (21–25)	-	-
Medicine	13 (11–15)	-	-
Dentistry	3 (2–4)	-	-
Pharmacy	5 (4–6)	-	-
Engineering	26 (24–28)	-	-
Environmental Science	10 (9–12)	-	-
Fisheries	6 (5–8)	-	-
Academic life			
Individuals with maladjustment (%)	29 (26–31)	-	-
Individuals with employment anxiety (%)	50 (48–53)	-	-

Notes: A numerical value shows the percentage of all participants which added IBS with the healthy person. Age data are expressed as mean ± standard deviation. D-IBS: IBS with diarrhea; C-IBS: IBS with constipation; M-IBS: Mixed IBS; U-IBS: unsubtyped IBS

Table 2 shows the relationship between positive IBS screening and the odds ratio for experiencing maladjustment in academic life. The crude odds ratio for maladjustment in academic life was significantly higher in students who screened positively for IBS relative to those who screened negatively (OR, 1.48; 95% CI, 1.15–1.90). After adjusting for age, sex, and faculty, the odds ratio for maladjustment in academic life was significantly higher in students who screened positively for IBS relative to those who screened negatively (OR, 1.62; 95% CI, 1.24–2.21).

**Table 2:** Relationship between IBS Status and Odds Ratios (ORs) for Experiencing Maladjustment in Academic Life.

IBS status	No. with maladjustment/ no. of participants	Crude odds ratio (A)		A + age and sex adjusted (B)		B + faculty adjusted (C)	
		OR (95% CI)	p-value	OR (95% CI)	p-value	OR (95% CI)	p-value
Negative screen for IBS	355/1,322	1.00 (referent)	< 0.01	1.00 (referent)	< 0.01	1.00 (referent)	< 0.01
Positive screen for IBS	120/341	1.48 (1.15–1.90)		1.61 (1.24–2.09)		1.62 (1.24–2.12)	

Table 3 shows the relationship between the subtypes of IBS and odds ratios for experiencing maladjustment in academic life. The crude odds ratio for maladjustment in academic life was significantly higher in students with D-IBS relative to students who screened negatively for IBS (OR, 2.00; 95% CI, 1.25–3.17). After adjusting for age, sex, and faculty, the odds ratios for maladjustment in academic life were significantly higher in students with D-IBS, C-IBS, and M-IBS relative to students who screened negatively for IBS (OR, 2.13; 95% CI, 1.31–3.43; OR, 2.00; 95% CI, 1.01–3.87; OR, 1.47; 95% CI, 1.01–2.06, respectively).

**Table 3:** Relationships between Subtypes of IBS Status and Odds Ratios (ORs) for Experiencing Maladjustment in Academic Life.

Subtypes of IBS Status	No. with maladjustment/ no. of participants	Crude odds ratio (A)		A + age and sex adjusted (B)		B + faculty adjusted (C)	
		OR (95% CI)	p-value	OR (95% CI)	p-value	OR (95% CI)	p-value
Negative screen for IBS	355/1322	1.00 (referent)		1.00 (referent)		1.00 (referent)	
Positive screen for IBS							
D-IBS	33/78	2.00 (1.25–3.17)	< 0.01	1.99 (1.24–3.18)	< 0.01	2.13 (1.31–3.43)	< 0.01
C-IBS	16/41	1.74 (0.90–3.27)	0.10	2.03 (1.04–3.87)	0.039	2.00 (1.01–3.87)	< 0.05
M-IBS	56/172	1.32 (0.93–1.84)	0.12	1.44 (1.01–2.03)	0.043	1.47 (1.01–2.06)	< 0.05
U-IBS	15/50	1.17 (0.61–2.12)	0.63	1.37 (0.69–2.46)	0.37	1.26 (0.65–2.34)	0.48

D-IBS: IBS with diarrhea; C-IBS: IBS with constipation; M-IBS: Mixed IBS; U-IBS: unsubtyped IBS

Table 4 shows the relationship between screening positively for IBS and the odds ratio for employment anxiety in academic life. The crude odds ratio for employment anxiety in academic life was significantly higher in students who screened positively for IBS relative to those who screened negatively (OR, 2.20; 95% CI, 1.72–2.83). After adjusting for age, sex, and faculty, the odds ratio for experiencing employment anxiety in academic life was significantly higher in students who screened positively for IBS relative to those who screened negatively (OR, 2.16; 95% CI, 1.68–2.81).

**Table 4:** Relationship between Having IBS and Odds Ratios (ORs) for Experiencing Employment Anxiety in Academic Life.

IBS status	No. with employment anxiety/ no. of participants	Crude odds ratio (A)		A + age and sex adjusted (B)		B + faculty adjusted(C)	
		OR (95% CI)	p-value	OR (95% CI)	p-value	OR (95% CI)	p-value
Negative screen for IBS	606/1322	1.00 (referent)	< 0.01	1.00 (referent)	< 0.01	1.00 (referent)	< 0.01
Positive screen for IBS	222/341	2.20 (1.72–2.83)		2.13 (1.66–2.76)		2.16 (1.68–2.81)	

Table 5 shows the relationship between subtypes of IBS and the odds ratio for experiencing employment anxiety in academic life. The crude odds ratios for employment anxiety were significantly higher in students with D-IBS, M-IBS, and U-IBS relative to students who screened negatively for IBS (OR, 1.79; 95% CI, 1.12–2.86; OR, 2.45; 95% CI, 1.75–3.43; OR, 3.04; 95% CI, 1.62–5.69, respectively). After adjusting for age, sex, and faculty, the odds ratios for employment anxiety were significantly higher in students with D-IBS, M-IBS, and U-IBS relative to students who screened negatively for IBS (OR, 1.85; 95% CI, 1.16–3.00; OR, 2.42; 95% CI, 1.72–3.44; OR, 2.93; 95% CI, 1.58–5.72, respectively).

**Table 5:** Relationships between Subtypes of IBS Status and Odds Ratios (ORs) for Experiencing Employment Anxiety in Academic Life.

Subtypes of IBS Status	No. with employment anxiety/ no. of participants	Crude odds ratio (A)		A + age and sex adjusted (B)		B + faculty adjusted(C)	
		OR (95% CI)	p-value	OR (95% CI)	p-value	OR (95% CI)	p-value
Negative screen for IBS	606/1322	1.00 (referent)		1.00 (referent)		1.00 (referent)	
Positive screen for IBS							
D-IBS	47/78	1.79 (1.12–2.86)	0.013	1.80 (1.13–2.90)	0.013	1.85 (1.16–3.00)	< 0.01
C-IBS	23/41	1.51 (0.81–2.82)	0.20	1.43 (0.76–2.72)	0.27	1.35 (0.72–2.58)	0.36
M-IBS	116/172	2.45 (1.75–3.43)	< 0.01	2.37 (1.69–3.35)	< 0.01	2.42 (1.72–3.44)	< 0.01
U-IBS	36/50	3.04 (1.62–5.69)	< 0.01	2.89 (1.57–5.60)	< 0.01	2.93 (1.58–5.72)	< 0.01

D-IBS: IBS with diarrhea; C-IBS: IBS with constipation; M-IBS: Mixed IBS; U-IBS: unsubtyped IBS

## Discussion

In this cross-sectional study, because the academic lives of students with IBS were reported to be less satisfactory than those reported by students without IBS, hypothesis 1 was supported. Furthermore, because employment anxiety was higher in students with IBS relative to that reported by those without, hypothesis 2 was also supported.

Two major reasons are assumed for the finding that the academic lives of students with IBS were less satisfactory than those of students without IBS. One of these reasons concerns the involvement of mental health problems. With respect to psychological abnormalities in IBS, there is some evidence that remarkable neuroticism is associated with the condition [12, 27, 28]. In a cross-sectional study of 655 university students in Japan, neuroticism was found to be significantly higher in students with IBS than in students without IBS [12]. Furthermore, high levels of depression have been observed in IBS patients [29, 30]. In an epidemiological survey of a population of 5,000 individuals aged 18–45 years in Sweden, it was shown that IBS patients’ mental health was significantly less satisfactory than that observed in healthy individuals [29]. A previous study showed that living in a dormitory while at school was a predictor for IBS in university students [19]. In addition, living in a dormitory while at school has been identified as one of the factors that contribute to the deterioration of mental health in university students [31]. That is, due to poor mental health in students with IBS in this study, their academic lives may have been significantly less satisfactory than those of students without IBS.

Another possibility is that the academic lives of students with IBS may have been less satisfactory than those of students without IBS because of the pain involved in the condition. One of the characteristic pathophysiological features of IBS is visceral hypersensitivity [5]. In a research study involving 49 IBS patients (mean age ± SD = 38 ± 15 years), the QOL of IBS patients who reported continuous abdominal pain was significantly lower than that of IBS patients without continuous abdominal pain [32]. Therefore, in university students with IBS, lower QOL resulting from experiencing continuous pain may have contributed to the deterioration of their academic lives.

One reason that employment anxiety was higher in students with IBS relative to those without, may be that psychological features of IBS include high anxiety and depression [33, 34]. IBS is often complicated by anxiety disorder [35, 36]. In an epidemiological survey of 13,537 American individuals, it was revealed that 7.2% of IBS patients also suffered from panic disorder [37]. High levels of state or trait anxiety in IBS university students may also have increased their employment anxiety.

There were four limitations to this study. First, this research only targeted students from one university, and prevalence rates for IBS vary according to culture [38, 39]. The target university is located in a medium-sized city on the island of Kyushu in western Japan. Therefore, it is unclear whether the results can be extrapolated to Japanese university students in general. Second, participants were categorized as IBS positive based on the results of a questionnaire rather than a physician’s diagnosis. Thus, the rate of IBS identified in this study may have been overestimated relative to IBS as diagnosed by a physician. If the prevalence rate for IBS found in this study is indeed higher than the prevalence rate that would have resulted from a physician’s diagnosis, the results may include data from individuals with relatively minor symptoms. If the study had only included patients with physician-diagnosed IBS, the relationship between psychological traits and IBS severity may have been more apparent; therefore, this relationship may have been underestimated in this study. Third, as this study was cross sectional, it was not possible to judge the influence of the presence of IBS or the degree of IBS symptom severity on the students’ academic lives. Fourth, existing scales, including the one used in this study, have not been adequately validated for use as academic life questionnaires in studies involving university students.

Clinically, in order to enhance scholastic support for university students with IBS, regular screening may be effective. In this study, we conducted an evaluation of academic life and IBS using questionnaires. By performing screening using a battery of tests, effects of advice to undergo medical examinations and early treatment can be expected.

In conclusion, academic life was less satisfactory and employment anxiety was higher in students with IBS relative to that observed in students without IBS. Because students with IBS experienced unsatisfactory academic lives, it is important to control IBS symptoms in this group.
